# Synthesis of radioactively labelled CdSe/CdS/ZnS quantum dots for in vivo experiments

**DOI:** 10.3762/bjnano.5.247

**Published:** 2014-12-10

**Authors:** Gordon M Stachowski, Christoph Bauer, Christian Waurisch, Denise Bargheer, Peter Nielsen, Jörg Heeren, Stephen G Hickey, Alexander Eychmüller

**Affiliations:** 1 Physical Chemistry, Technische Universität Dresden, 01062 Dresden, Germany; 2 Li-Tec Battery GmbH, Am Wiesengrund 7, 01917 Kamenz, Germany; 3 Department of Biochemistry and Molecular Cell Biology, University Medical Centre Hamburg-Eppendorf, 20246 Hamburg, Germany; 4 School of Chemistry and Forensic Science, University of Bradford, Richmond Road, Bradford BD7 1DP, United Kingdom

**Keywords:** biomarker, CdSe/CdS/ZnS, quantum dots, radioactive labelling, ^65^Zn

## Abstract

During the last decades of nanoparticles research, many nanomaterials have been developed for applications in the field of bio-labelling. For the visualization of transport processes in the body, organs and cells, luminescent quantum dots (QDs) make for highly useful diagnostic tools. However, intercellular routes, bio-distribution, metabolism during degradation or quantification of the excretion of nanoparticles, and the study of the biological response to the QDs themselves are areas which to date have not been fully investigated. In order to aid in addressing those issues, CdSe/CdS/ZnS QDs were radioactively labelled, which allows quantification of the QD concentration in the whole body or in ex vivo samples by γ-counting. However, the synthesis of radioactively labelled QDs is not trivial since the coating process must be completely adapted, and material availability, security and avoidance of radioactive waste must be considered. In this contribution, the coating of CdSe/CdS QDs with a radioactive ^65^ZnS shell using a modified, operator-safe, SILAR procedure is presented. Under UV illumination, no difference in the photoluminescence of the radioactive and non-radioactive CdSe/CdS/ZnS colloidal solutions was observed. Furthermore, a down-scaled synthesis for the production of very small batches of 5 nmol QDs without loss in the fluorescence quality was developed. Subsequently, the radio-labelled QDs were phase transferred by encapsulation into an amphiphilic polymer. γ-counting of the radioactivity provided confirmation of the successful labelling and phase transfer of the QDs.

## Introduction

Two decades of research and investigation in the field of luminescent semiconductor nanoparticles, also known as quantum dots (QDs), have now passed since the fundamental work of Bawendi and co-workers, which focused particularly on the synthesis of II–VI materials [1–5]. CdSe-based, state-of-the-art synthetic protocols typically use the hot injection method [1] and can deliver QDs with visible emission which possess quantum yields (QYs) of up to 85% as core/shell or alloyed structures [5–8]. Semiconductor nanocrystals are discussed in the literature as potential emitters for LEDs or as bio-labelling agents due to their complementary optical properties as well as other advantages in comparison to organic dyes. For example, they exhibit broad absorption (allowing excitation energies higher than the band gap energy to be freely selected), narrow emission spectra, long emission lifetimes, photo- and chemical-stability, etc. [5,9–10].

Although they offer many advantages, it is important to evaluate the toxicity [11] of nano-scale materials, particularly for biological applications and to comply with the necessary safety and regulatory aspects. One main issue is their chemical toxicity due to their composition, as QDs with high QYs often contain Cd^2+^ or Hg^2+^, and their metabolism results in the release of toxic ions. This work represents a contribution to the investigation of the intercellular route taken by QDs, the quantification of the degradation or excretion of the nanoparticles, and the study of the biological response to QDs.

To address those issues, a methodology for radioactively labelling of QDs was developed. Applying this strategy enables the quantification of the QD concentration during cellular uptake to be calculated. Here, the measured amount of radioactivity can be related to the amount or concentration of the QD ensemble or their metabolites (i.e., ionic species). To avoid any potential chemical inconsistencies we replace the typical precursors present in the synthesis with their equivalent radionuclides. In the past, radionuclides such as ^109^Cd or ^111^In were used for intrinsic radiolabeling [12–13]. In this work, the Zn component of CdSe/CdS/ZnS core/shell/shell QDs was selected for replacement with ^65^Zn due to its ease of availability. Furthermore, due to the frequency with which ZnS is used as a shell material, the selection of ^65^Zn allows the variation of the core, offering a higher degree of versatility for the method.

The well-investigated CdSe-based QDs were utilised as a model system onto which CdS and ZnS are coated via the successive ion layer adsorption and reaction method (SILAR) in order to achieve greater chemical stability, an enhancement of the fluorescence QY and a shift in the photoluminescence (PL) wavelength to the therapeutic spectral window [3,6,8].

When developing a synthesis for radioactively labelled QDs, one must take into account the handling under typical chemical laboratory conditions with respect to both the safety aspects and material availability. Moreover, the common chemical laboratory synthesis batch size must be scaled down to avoid excessive radioactive waste while ensuring a statistically relevant probability of adequate labelling. Finally, for in vivo experiments the radioactively labelled QDs must be quantitatively and qualitatively transferred into the aqueous phase.

## Results and Discussion

### Radioactive synthesis

**Figure 1:**
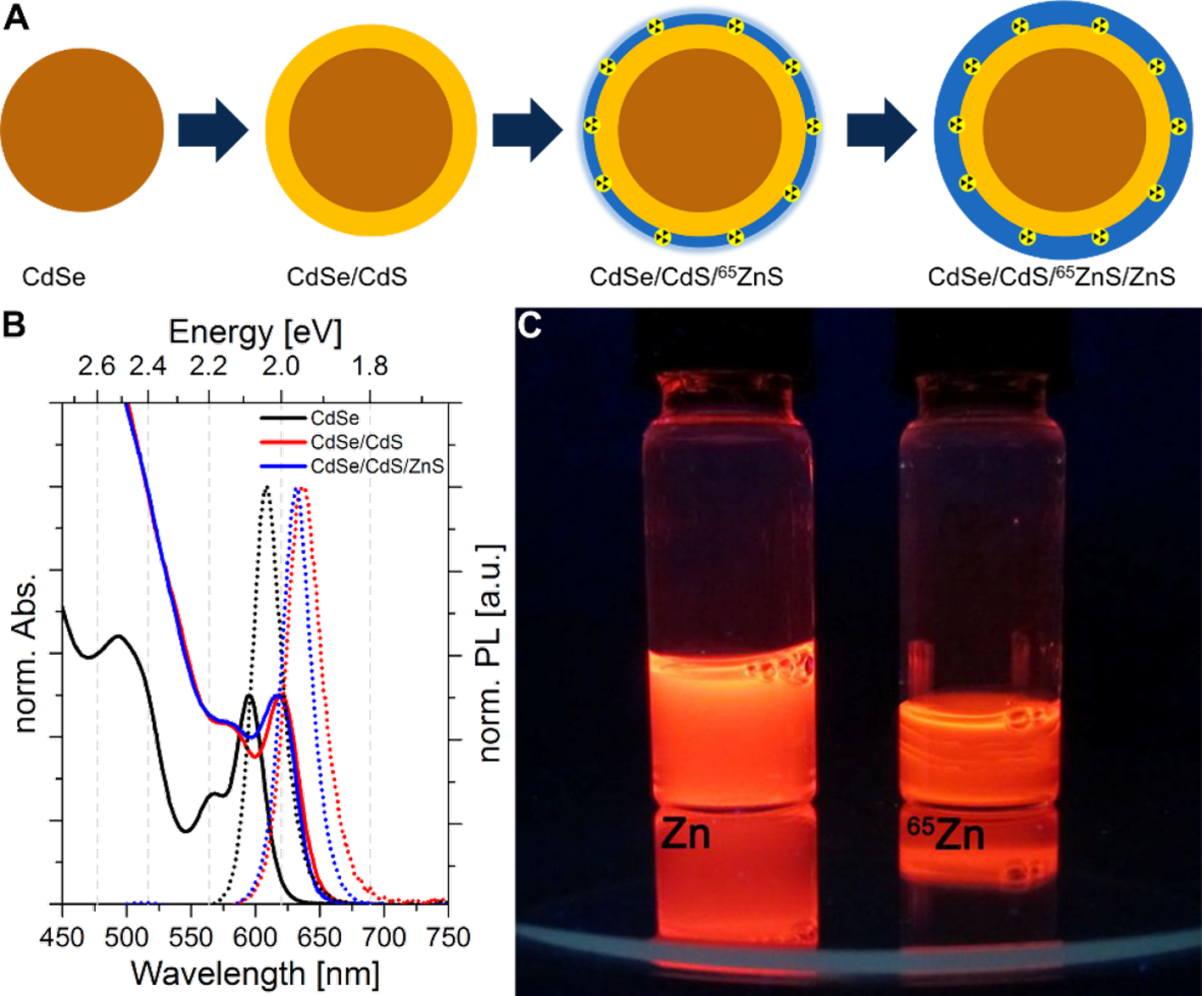
(A) Principle steps in the QD synthesis starting with the CdS coating of CdSe QDs and followed by stepwise application of ^65^ZnS and ZnS shells. (B) Absorbance (solid) and PL (dotted) spectra of CdSe, CdSe/CdS and non-radioactive CdSe/CdS/ZnS QDs. (C) Colloidal QD solutions of non-radioactive CdSe/CdS/ZnS (left) and radioactive CdSe/CdS/^65^ZnS/ZnS (right) under illumination by a UV lamp.

For the synthesis of radioactively labelled material, it was necessary to significantly alter the coating process from the routine SILAR protocol. The main challenges were to use ^65^ZnCl_2_ as the radioactive zinc precursor instead of the commonly used ZnO or Zn(oleate)_2_ in the SILAR synthetic routes and to reduce the contact time involved in the handling of the radioactive material as much as possible. For this purpose, ^65^ZnCl_2_ diluted in 0.1 M HCl_(aq)_ was placed in an empty, lead glass shielded flask where it was converted to a species more useful for the labelling of CdSe/CdS QDs by the in situ formation of zinc stearate (^65^Zn(stearate)_2_). After addition of the previously prepared QD materials and adsorption of ^65^Zn^2+^ onto the QD surface, the sulphur precursor was added to the flask to form a shell of ^65^ZnS. To avoid later desorption of radioactive ^65^Zn^2+^ ions from the surface, another ZnS shell (using a nonradioactive zinc precursor) was applied to the QDs using the typical SILAR procedure (Figure 1A) [6,8]. The main advantage of this method is that the radioactive material can be added at ambient temperature and shielded by lead glass using a pipette, which prevents direct hand contact from the very beginning. All other materials may be added safely from a sufficient distance and behind lead glass.

A red shift in the absorbance and PL spectra indicates the formation of a CdS shell surrounding the CdSe QDs (Figure 1B). During the formation of the non-radioactive ZnS shell, a slight blue shift and a weakening of the absorbance was observed. Due to the radioactivity and safety issues, spectra could not be taken for the CdSe/CdS/^65^ZnS/ZnS QDs. However, the PL of both the cleaned CdSe/CdS/ZnS and the CdSe/CdS/^65^ZnS/ZnS QDs show no significant difference under UV illumination (Figure 1C) with the PL remaining unchanged even after one month. The radiation due to the ^65^Zn decay does not appear to influence the PL intensity to any great extent.

**Figure 2:**
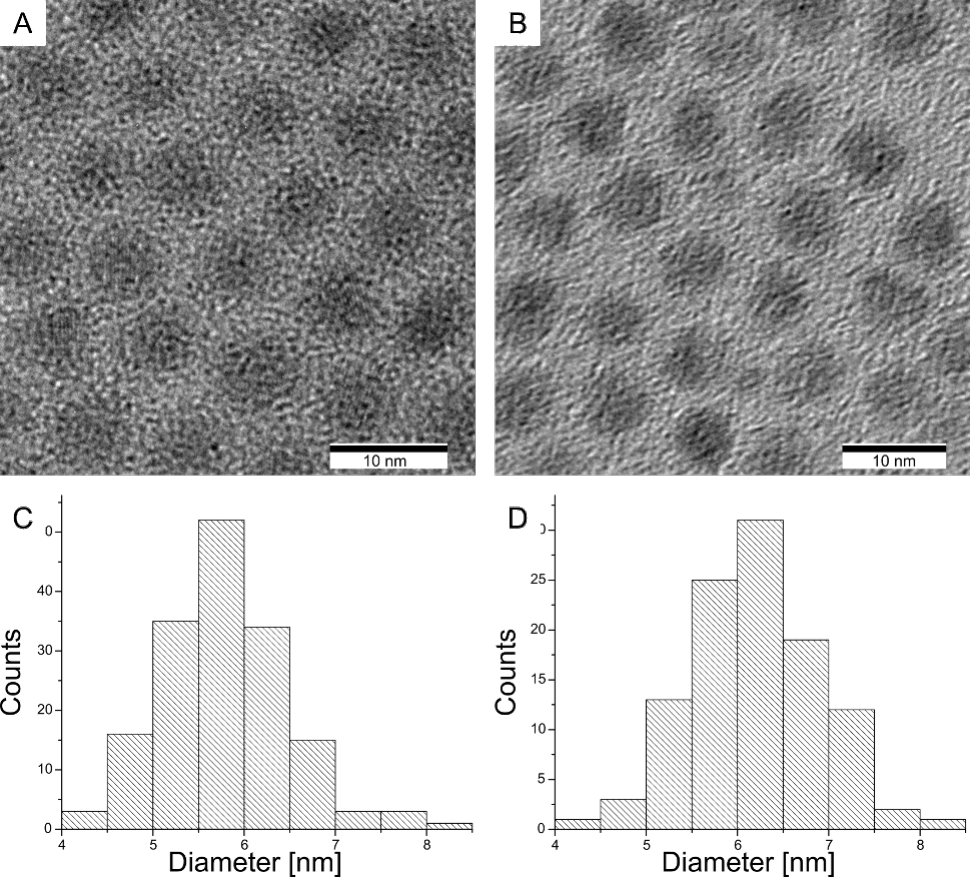
TEM images and associated size histograms of the CdSe/CdS QDs (A,C) and CdSe/CdS/ZnS (B,D) which show the diameter of the QDs to be 5.8 nm ± 0.7 nm and 6.4 nm ± 0.7 nm, respectively. Scale bars are 10 nm.

By quantification of the radioactivity of the ^65^Zn-containing QDs it was found that only 50% of the original ^65^Zn was detected in the cleaned QD solution, the rest being accounted for by the waste reaction and purification solution. Moreover, the non-radioactive QDs were investigated using TEM (Figure 2) and interestingly, instead of an expected size increase [8] of two monolayers of ZnS (≈1.2 nm), a nominal increase of only one monolayer was observed. Both effects point to an incomplete coating of the ZnS shells but the exact reason has yet to be determined.

### Minimization

**Figure 3:**
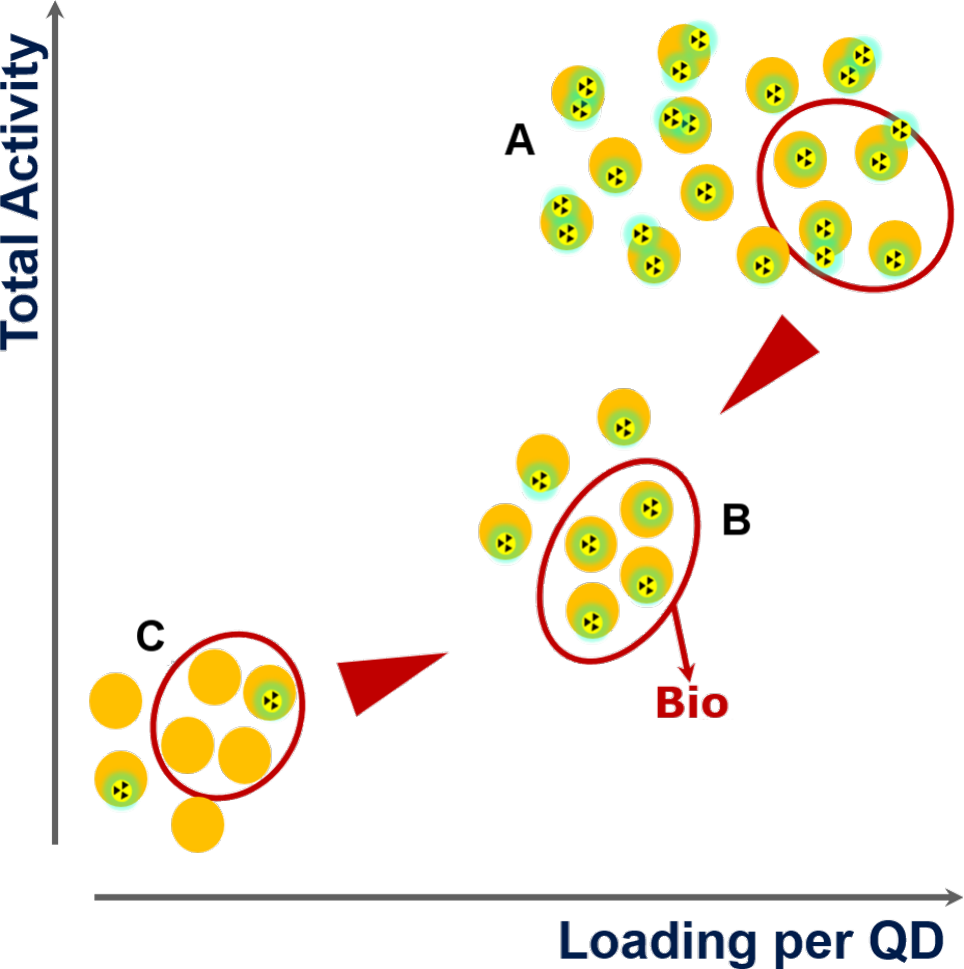
(A) shows excessive labelling and a large quantity of QDs which is not necessary for bio-applications. It is desirable to decrease the total amount of radioactivity (A → B) by reducing the excessive labelling (diminish the per-particle-loading) and the total amount of QDs simultaneously. (C) represents the presence of non-labelled QDs and should be avoided.

The amount of radioactive material that is required in order to produce a viable amount of material per batch is another very important aspect in the development of the synthesis. The amount of QDs typically produced on the lab scale via a hot injection process far exceeds the desired requirements for most biological experiments (Figure 3A). From the point of view of safety during the synthesis and subsequent bio-applications, the amount of radioactively labelled material should be kept to a minimum. Similarly, from an environmental perspective, in order to avoid radioactive waste, one must work with as small a batch as possible. Furthermore, while overloading the QDs with radioactive material should be avoided, the final synthesis design should guarantee an adequate degree of radioactivity per particle in order to avoid the statistical probability that an unnecessarily large amount of QDs remain unlabelled (B instead of C in Figure 3).

In light of the above considerations, down-scaling entails the design of a completely new synthesis route which includes the optimization of the typical synthesis parameters such as batch size, relative concentrations, injection and ripening temperatures, etc. By contrast, due to the extremely small volumes and masses involved, the down-scaling is limited by the degree of control over the stirring rates and temperature. A judicious modification of the protocol with respect to the ratio of reactants, ligands and solvents allows the synthesis procedure to be scaled down to amounts of CdSe/CdS QDs on the order of 5 nmol.

Furthermore the procedure has been designed to enable the operator to vary the ratio between ^65^ZnS and ZnS in the inner shell, by addition of non-radioactive ZnCl_2_ or Zn(stearate)_2_, in order to be able to attain a predefined ratio, thus allowing one to choose the specific radioactivity in the ^65^ZnS (inner) shell. For example, in a typical 5 nmol batch with a desired final radioactivity of 5 µCi, it was possible to choose the ratio of ^65^ZnCl_2_ and ZnCl_2_ to be very close to 1:10. By comparison, a single application of co-labelled QDs in in vivo experiments demands 1 µCi per mouse.

### Phase transfer

The synthesis of CdSe/CdS/^65^ZnS/ZnS QDs was carried out in organic media. However, to extend their potential range of application, the nanoparticles should be dispersible in biologically compatible, aqueous media. Therefore, an encapsulation and phase transfer method described by Shtykova et al. was used to introduce the QDs, stabilized by nonpolar ligands, into an amphiphilic polymer [14]. Under UV illumination, similar emission colours for both, the hydrophilic, encapsulated and the hydrophobic QDs were detected, which is evidence for the success of the encapsulation as well as the fact that nanoparticles remain uncompromised during the wrapping procedure. However, it was found by γ-counting of radioactivity that a dramatic decrease of about 90% occurred during the encapsulation step. Initial studies suggest that the decrease in radioactive activity during the encapsulation and phase transfer steps appears to be due to the harsh ultra-sonication of the QDs during the procedure. The possibility of employing less energy intensive methods such as shaking or stirring is currently under investigation.

## Conclusion

A method for the labelling of CdSe/CdS/ZnS QDs during the synthesis process with radioactive ^65^Zn in one of the outer shells of the nanoparticles has been developed. The challenges associated with the handling of radioactive materials and the minimization of the synthesis in order to avoid excessive generation of radioactive waste have been resolved. To this effect, a procedure strategy was designed where the main advantage is a high degree of operator safety and the avoidance of direct hand contact with the radioactive materials. Further, by varying the amounts of ^65^ZnCl_2_ and ZnCl_2_, the radioactivity per particle can be controlled. Interestingly, we observed a ^65^ZnS shell formation which was thinner than expected the thickness, resulting in a lower ^65^Zn incorporation, the exact origin of which is currently under investigation.

The process of encapsulation and phase transfer causes a high loss in radioactivity, which is suspected to be due to the harsh sonication conditions. Quantification of the QD solution via absorption and PL measurements should help to understand the mechanism involved during this step. Phase transfer strategies should be pursued in order to allow comparison of differently transferred QDs for their in vivo response.

Owing to its decay, ^65^Zn is transformed into Cu and therefore over time the amount of CuCl_2_ present in the ^65^ZnCl_2_ containing 0.1 M HCl_(aq)_ increases. It is clear that when CuCl_2_ is present in the initial reaction mixture it can be transformed to Cu(stearate)_2_ during the transformation [15] of the ^65^ZnCl_2_, however, the degree of transformation is unknown. The decay of ^65^Zn occurs in the QDs and the influence of Cu in the coating layer of the QDs should be more fully investigated in terms of its lattice mismatch, self-purification and optical properties (e.g., dopant emission, PL enhancement or quenching).

## Experimental

### Synthesis of CdSe/CdS QDs

Into a 25 mL three-neck flask, 10 mL of 1-octadecene (ODE), 0.4 mmol of Cd(oleate)_2_ as a stock, 2 g hexadecylamine and 2 g trioctylphosphine oxide was loaded. After degassing, the temperature was set to 270 °C and 0.4 mmol of trioctylphosphine selenide (TOP:Se) as a stock was injected rapidly under an Ar flow. After remaining for 20 min at 245 °C, the reaction was quenched with toluene and the CdSe QDs were purified.

100 nmol of the purified QDs (4.3 nm), 1.5 g of octadecylamine (ODA) and 6 mL of ODE were placed in a 25 mL three-neck flask and degassed. At 220 °C, cation and anion precursors, Cd(oleate)_2_ and S as an ODE stock solution (ODE:S), were added alternating with waiting times of 5 and 25 min after each injection, respectively. The amounts were calculated based on the addition of two times one monolayer for the given size of the CdSe QDs.

### Synthesis of CdSe/CdS/^65^ZnS/ZnS QDs

The following specifications are calculated for a certain radioactivity of ^65^ZnCl_2_ containing 0.1 M HCl, namely, 556 µg ^65^Zn/(mL HCl) and 122 µCi/(mL HCl). A 25 mL three-neck flask shielded with lead glass was loaded with 4.0 µmol of ZnCl_2_ (92% of total ZnCl_2_) diluted in methanol and 41.2 µL of a ^65^ZnCl_2_/HCl solution (22.9 µg ^65^Zn, 0.35 µmol ^65^Zn, 8% of total ZnCl_2_, 5 µCi). The HCl and H_2_O were then evacuated from the flask. A mixture of 30.6 µmol stearic acid and 17.5 µmol tetramethylammonium hydroxide in methanol was added dropwise. The precipitated ^65^Zn(stearate)_2_ was then dried under vacuum. For the particle coating, as adopted from Li et al. [6], 5 nmol of the previously prepared CdSe/CdS QDs (5.7 nm), 50 mg of ODA and 2 mL of ODE were added and the solution was placed under inert gas atmosphere. Under room temperature, 50 µL of a 0.1 M ODE:S stock solution was added and the temperature was set to 220 °C for the coating step. After 30 min, 60 µL of a 0.1 M Zn(oleate)_2_, and 35 min thereafter 100 µL of 0.1 M ODE:S, were added dropwise at 200 °C. After 60 min the reaction was quenched with 2 mL of toluene. In comparison to typical clean-up procedures for non-radioactive QDs, only two precipitation steps were performed behind lead glass to purify radioactive QDs to avoid large amounts of radioactive waste. The amount of ^65^ZnCl_2_ solution and other components used for the coating must be adjusted with respect to the concentration and size [8] of the CdSe/CdS QDs that are used. Radioactive waste was temporarily stored and collected by an authorized waste management enterprise under appropriate safety precautions.

### Measurements

For absorption and emission spectroscopy, a Cary 50 Scan (Varian) and a Fluoromax 4 (Horiba), respectively, were used. TEM was performed on a Libra 200 (Zeiss) instrument. For radioactivity analysis, the Hamburg Whole Body Radioactivity Counter [16] was used to measure ^65^Zn radioactivity.
